# Prophylactic metformin after antenatal corticosteroids (PROMAC): a double blind randomized controlled trial

**DOI:** 10.1186/s12884-021-03628-5

**Published:** 2021-02-15

**Authors:** Jesrine Gek Shan Hong, Peng Chiong Tan, Maherah Kamarudin, Siti Zawiah Omar

**Affiliations:** Department of Obstetrics and Gynecology, Faculty of Medicine, University of Malaya, Lembah Pantai, 50603 Kuala Lumpur, Malaysia

**Keywords:** Antenatal corticosteroids, Blood sugar profile, Gestational diabetes, Hyperglycemia, Metformin, Premature labor

## Abstract

**Background:**

Antenatal corticosteroids (ACS) are increasingly used to improve prematurity-related neonatal outcome. A recognized and common adverse effect from administration of antenatal corticosteroid is maternal hyperglycemia. Even normal pregnancy is characterized by relative insulin resistance and glucose intolerance. Treatment of maternal hyperglycemia after ACS might be indicated due to the higher risk of neonatal acidosis which may coincide with premature birth. Metformin is increasingly used to manage diabetes mellitus during pregnancy as it is effective and more patient friendly. There is no data on prophylactic metformin to maintain euglycemia following antenatal corticosteroids administration.

**Methods:**

A double blind randomized trial. 103 women scheduled to receive two doses of 12-mg intramuscular dexamethasone 12-hour apart were separately randomized to take prophylactic metformin or placebo after stratification according to their gestational diabetes (GDM) status. First oral dose of allocated study drug was taken at enrolment and continued 500 mg twice daily for 72 hours if not delivered. Six-point blood sugar profiles were obtained each day (pre- and two-hour post breakfast, lunch and dinner) for up to three consecutive days. A hyperglycemic episode is defined as capillary glucose fasting/pre-meal ≥ 5.3 mmol/L or two-hour post prandial/meal ≥ 6.7 mmol/L. Primary outcome was hyperglycemic episodes on Day-1 (first six blood sugar profile points) following antenatal corticosteroids.

**Results:**

Number of hyperglycemic episodes on the first day were not significantly different (mean ± standard deviation) 3.9 ± 1.4 (metformin) vs. 4.1 ± 1.6 (placebo) *p* = 0.64. Hyperglycemic episodes markedly reduced on second day in both arms to 0.9 ± 1.0 (metformin) vs. 1.2 ± 1.0 (placebo) *p* = 0.15 and further reduced to 0.6 ± 1.0 (metformin) vs. 0.7 ± 1.0 (placebo) *p* = 0.67 on third day. Hypoglycemic episodes during the 3-day study period were few and all other secondary outcomes were not significantly different.

**Conclusions:**

In euglycemic and diet controllable gestational diabetes mellitus women, antenatal corticosteroids cause sustained maternal hyperglycemia only on Day-1. The magnitude of Day-1 hyperglycemia is generally low. Prophylactic metformin does not reduce antenatal corticosteroids’ hyperglycemic effect.

**Trial registration:**

The trial is registered in the ISRCTN registry on May 4 2017 with trial identifier 10.1186/ISRCTN10156101.

## Background

Antenatal corticosteroids (ACS) are commonly used in obstetric practice to reduce the incidence of prematurity-related neonatal morbidity and mortality [1–3]. Antenatal dexamethasone and betamethasone use had similar survival free of neurosensory disability at age 2 years [4]. In our center, the standard ACS regime is two doses of dexamethasone 12-mg 12 hours apart, an ACS regime also widely used in the whole of Malaysia [5].

A widely recognized and common side effect from administration of ACS is maternal hyperglycemia [6]. Even normal pregnancy is characterized by relative insulin resistance and glucose intolerance [7]. Hyperglycemia occurs in a majority of patients after ACS-betamethasone regardless of diabetes status [8]. In gestational diabetes mellitus (GDM) or pre-existing diabetes mellitus, ACS will result in deterioration of glycemic control, severe hyperglycemia and increase in insulin requirement [9]. The transient maternal hyperglycemia in women without diabetes after ACS-betamethasone can be limited by the concurrent use of insulin [6]. Initiation of insulin therapy or increase in insulin dosage may be required for glycemic management after ACS [10].

Hyperglycemia after ACS can have an effect on fetal acid-base status which may coincide with preterm delivery: glucose compared with saline intravenous infusion to laboring women with ketonuria significantly decreases fetal blood pH [11] and maternal glucose infusion just prior to delivery results in maternal hyperglycemia and subsequent fetal acidosis [12, 13].

Multiple international guidelines state that metformin may be used as an alternative or adjunct during pregnancy [14–16] to control hyperglycemia. Metformin improves insulin sensitivity, reduces hepatic gluconeogenesis, increases peripheral glucose uptake [17, 18], and is not associated with an increase in hypoglycemia [19]. The onset of metformin is about three hours with peak plasma concentrations (C_max_) reached within one to three hours of taking immediate release metformin [20] and this may reduce the hyperglycemic effect after receiving ACS. The oral route of administration helps to improve patient compliance.

We hypothesized that prophylactic metformin concomitant with ACS will reduce the expected transient ACS-dexamethasone induced hyperglycemia.

## Methods

The trial was carried out in the Obstetric unit of the University Malaya Medical Center, Kuala Lumpur, Malaysia between May 25 and November 17 2017.

### Participants

Inclusion criteria were women age ≥ 18 years old, singleton, 24–38 weeks’ gestation, about to receive or within 6 hours of first dose of dexamethasone (12 mg for two doses 12 hours apart regimen – our institutional standard ACS protocol [5]). These women were stratified to diet controlled GDM and euglycemic pregnancies before separate randomization to trial intervention. GDM is defined in our population as 75 g oral glucose tolerance test (OGTT) fasting blood glucose ≥ 5.1mmol/L and /or 2-hour post-prandial glucose ≥ 7.8mmol/L in pregnant women without prior history of hyperglycemia based on the Malaysian national clinical practice guideline [21]. Patients on any hypoglycemic agent, pre-existing diabetes mellitus, baseline capillary blood glucose level more than 11mmol/L (at recruitment), in active labor or likely to deliver within the next 24 hours after administration of ACS, suspected chorioamnionitis, maternal or fetal infection, on beta-sympathomimetic agent tocolysis or on diet restrictions in anticipation of imminent (within 24 hours) birth by Cesarean section were excluded.

### Recruitment and randomization

Eligible women were approached, provided with patient information sheet and verbally counseled with regards to trial participation by the co-investigator (JGSH) or care provider. Written informed consent was obtained from every participant. Capillary blood glucose was tested at recruitment (random baseline) and women excluded if this value exceeds 11mmol/L. Participants received intramuscular dexamethasone, 12 mg for two doses 12 hours apart, as per our institution ACS protocol. All participants’ relevant demographic and clinical data were transcribed onto the Case Report Form.

Participants were stratified to euglycemic women and women with diet-controlled GDM for separate randomization to either use of metformin or placebo on 1 to 1 ratio by opening the lowest numbered available, sealed and opaque envelope. Envelopes were prepared based on a computer-generated (using random.org) random sequence in random blocks of two or four, which contained the study drug (a pack of six 500 mg metformin tablets or identical placebo) by a co-investigator (PCT) who was not involved in the trial recruitment.

### Interventions

 Randomized participants were observed to take the first dose of their allocated study (500 mg metformin or identical placebo tablet) at enrolment under direct supervision with the remaining five tables to be taken twice daily, which was at their next breakfast or dinner. If the second dose (at the next breakfast or dinner) was within 6 hours of the first dose, it would be omitted, with the second dose to be taken at the subsequent breakfast or dinner. After delivery if it occurred within the 3 days’ study period, the study drug was stopped. Both participants and investigators were masked.

### Post intervention care

 All participants were taught how to use their personal glucometer [Abbott, Freestyle Freedom Lite®] started from the baseline measurement prior to recruitment and supplied with the necessary materials for self-monitoring of their blood sugar profiles (BSP). All participants were instructed to perform their first trial blood sugar reading before their next meal or 2-hour after their last meal. Participants are then to continue their 6 points per day BSP monitoring, pre and post the three main meals for the next three days (for a maximum total of 18 BSP points) if they remained undelivered and to record the values on the study BSP diary.

Participants were instructed to continue with their usual diet during the study period. Participants could be discharged within the 3 days’ study period if clinically appropriate but would continue with BSP monitoring and allocated intervention. If a BSP reading > 11mmol/L during monitoring, providers or discharged participants were instructed to contact co-investigator (JGSH). JGSH contact number was also given to all discharged participants to seek advice as needed. Open label 500 mg metformin tablets were provided to all participants in a separate sealed pack labeled as “Rescue Metformin, Use Only as Instructed”. The open label rescue metformin tablets were obtained from a different manufacturer [Glucophage 500 mg film coated tablet, Merck Serono Limited] and visually distinct from the study metformin or placebo identical tablets. Participants with blood glucose level > 11mmol/L whether pre or post-prandial were usually instructed to take an additional open label 500 mg metformin. A maximum of three open label 500 mg metformin was taken per day (maximum intake of 2500 mg per day for metformin arm). In the event where glycemic control was considered insufficient, subcutaneous human insulin was used (0.2 u/kg) with re-hospitalization if the participant was already discharged.

### Outcome measures

A hyperglycemic episode was defined as capillary blood glucose fasting or pre-meal of ≥ 5.3mmol/L or 2 hours post-meal ≥ 6.7mmol/L[16, 21, 22]. A hypoglycemic episode (Level 1) is defined as capillary blood glucose ≤ 3.9mmol/L irrespective of symptoms[23]. Compliance with monitoring was defined as at least 4 BSP points being available of each 6-point BSP blocks each 24 hours. Non-compliant (3 or less BSP point available) participants were excluded from analysis of that 6 points BSP block.

The study BSP diary was obtained from the participant after completion of BSP monitoring over three days or earlier if delivered. Participants were instructed to self-chart any symptoms of nausea, vomiting or diarrhea on participants’ diary. Satisfaction score (numerical rating scale NRS 0 to 10, higher score, greater satisfaction) with the glucose monitoring and study drug treatment were obtained after three days of BSP monitoring or as soon as possible after delivery if had occurred. The participants’ hospital notes were retrieved after delivery and relevant study outcomes were transcribed onto the Case Report Form. Newborn outcomes were similarly obtained.

The primary outcome was the number of hyperglycemic episodes in the first 24 hours (first 6 BSP points) following first intramuscular dose of 12 mg dexamethasone.

Pre-specified secondary outcomes were hyperglycemic episodes in the second and third 24 hours after administration of antenatal dexamethasone (if still undelivered), hypoglycemic episodes (hypoglycemia is defined as capillary blood glucose level ≤ 3.9mmol/L[16, 21, 22]), need for additional or unplanned antiglycemic agent (metformin or insulin) as indicated by capillary blood glucose level ≥ 11mmol/L, nausea, vomiting or diarrhea (throughout study period up to Day 3 or delivery), maternal outcomes (mode of delivery and estimated blood loss during delivery), neonatal outcomes (birthweight, umbilical cord arterial pH and base excess at birth, Apgar score at 1 and 5 minute, special care nursery/neonatal intensive care unit admission during birth admission) and patient satisfaction NRS score with their allocated study drug regimen.

### Sample size calculation

The sample size was calculated by assuming a difference of 1 and a standard deviation of 1 in number of hyperglycemic episodes in the first 24-hours (comprised 6 capillary glucose readings), utilizing alpha of 0.05 and power of 90 %, 22 participants would be needed in each arm. If the Mann Whitney U test was used rather than Student t test (if data value distribution is non-normal; 10 % sample size increase), up to 15 % drop-outs [22/(0.9 × 0.85) = 28.8] and rounding up, 30 participants were needed in each arm (N = 60) for a powered trial.

### Statistical analysis

Data were entered into a statistical software package SPSS (Version 23, IBM© SPSS© Statistics) by co-investigator (JGSH). The Student’s t test was used to analyze means and distributions. Chi-square test was used for categorical datasets. Mann-Whitney U test for non –normally distributed data. P value < 0.05 was regarded as significant. Analysis was on intention-to-treat basis (incomplete data sets were excluded).

### Ethical aspects

Women who chose not to participate received standard care and participants who decided to withdraw were able to do so without having to give a reason and their care was not affected. Participants were not remunerated.

## Results

**Fig. 1:**
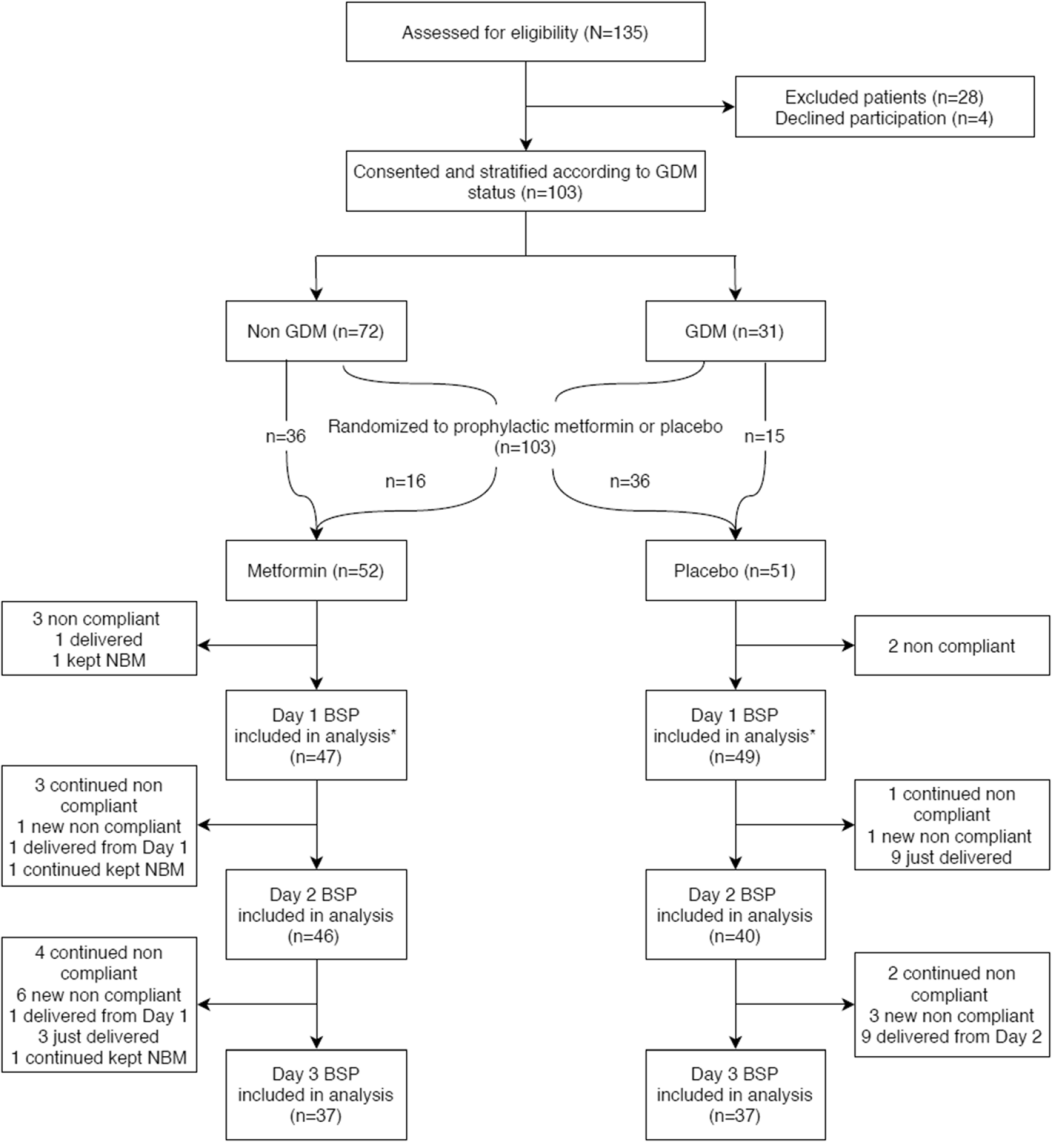
Recruitment flow chart into a double-blind randomized trial of prophylactic metformin or placebo after antenatal corticosteroids

Figure 1 depicts the flow of participants into the study and dropouts throughout the 3-day study period. After the original sample size target of at least 60 (48 non-GDM and 18 GDM women enrolled) was achieved, we opted to extend recruitment to 100 women to increase numbers in GDM subgroup (n ≥ 30) for better power in subgroup statistical analyses. A total of 135 women who fulfilled the inclusion criteria were approached but only 103 were recruited and randomized. The 28 women excluded were for the following reasons 9 were on diet restriction in anticipation for Cesarean birth, 7 were multiple pregnancies, 7 were already on antiglycemic agent such as metformin and/or insulin, 3 were pre-existing diabetes mellitus, 2 were in active labor and 4 declined participations. All 103 participants provided written consented and their recruitment capillary blood glucose levels were taken. After stratification, there were 72 non-GDM and 31 GDM participants. 52 participants (36 non-GDM, 16 GDM women) were randomized to metformin and 51 (36 non-GDM and 15 GDM women) to placebo. All participants completed their antenatal 2-dose dexamethasone course. Complete 18-points BSP were available in 33 and 32 participants in metformin and placebo arms respectively.

Table 1 shows the characteristics of the trial participants dichotomized according to assignment to prophylactic metformin or placebo trial arms. Characteristics as expected were similar across the trial arms. The first blood sugar profile point recorded by participants after receiving first dose of dexamethasone were analyzed and did not show significant difference.

**Table 1:** Characteristics of trial participants stratified to treatment allocation (prophylactic metformin or placebo) after antenatal corticosteroids

	Metformin *n* = 52	Placebo *n* = 51	*p* value
Age (years)	31.3 ± 4.4	31.0 ± 3.4	0.71
Recruitment capillary glucose (mmol/L)	5.1 ± 0.8	5.0 ± 0.9	0.85
Recruitment gestational age (weeks)	33.7 [32.1-35-1]	34.7 [32.6–36.4]	0.11
Nulliparous	27 (51.9 %)	26 (51.0 %)	0.92
Height (m)	1.57 ± 0.05	1.57 ± 0.04	0.99
Weight (kg)	72.2 ± 13.9	70.6 ± 14.7	0.55
Body mass index (kg/m^2^)	29.2 ± 5.6	28.4 ± 5.5	0.48
*Gestational diabetes*			0.88
Non-GDM	36 (69.2 %)	36 (70.6 %)	
GDM	16 (30.8 %)	15 (29.4 %)	
*Indication for antenatal dexamethasone*			0.82
Suspected preterm labor	16 (30.8 %)	14 (27.5 %)	
Antepartum hemorrhage	10 (19.2 %)	13 (25.5 %)	
Non-reassuring fetal status	13 (25.0 %)	10 (19.6 %)	
Others^a^	13 (25.0 %)	14 (27.5 %)	
* Ethnicity*			0.65
Malay	35 (67.3 %)	34 (66.7 %)	
Chinese	7 (13.5 %)	4 (7.8 %)	
Indian	6 (11.5 %)	6 (11.8 %)	
Others^b^	4 (7.7 %)	7 (13.7 %)	
1st dose dexamethasone to study drug (hours)	4.5 [3.0-5.5]	4 [2-5]	0.22
*First blood sugar profile point within trial*	n = 48	n = 50	
Before breakfast	5 (10.4 %)	2 (4.0 %)	0.10
2-hour after breakfast	5 (10.4 %)	6 (12.0 %)	
Before lunch	8 (16.7 %)	6 (12.0 %)	
2-hour after lunch	14 (29.2 %)	6 (12.0 %)	
Before dinner	9 (18.8 %)	18 (36 %)	
2-hour after dinner	7 (14.6 %)	12 (24.0 %)	

Data expressed as mean ± standard deviation, median [interquartile range] and number (%). Analyses by Student t test for comparison of means for normally distributed continuous data and by Mann-Whitney U test for non-parametric or ordinal data and Chi Square test for categorical data

^a^Other indications: 11 maternal hypertension, 9 planned Cesarean section, 5 preterm prelabor rupture of membrane, 1 reduced fetal movement, 1 pulmonary embolism

^b^Other ethnicities: 2 Sabah native, 4 Vietnamese, 1 Filipino, 1 Pakistani, 1 Yemeni, 1 Indonesian and 1 Syrian

Table 2 reports the primary outcome and secondary glycemia-based outcomes dichotomized according to assignment to metformin or identical placebo trial arms. The mean and standard deviation of hyperglycemic episodes in the first 24 hours after administration of dexamethasone showed no significant difference between metformin and placebo arm with 3.9 ± 1.4 (metformin) vs. 4.1 ± 1.6 (placebo) p = 0.64. There was no hypoglycemia noted in the first 24 hours in the metformin arm while placebo arm showed a mean of 0.1 ± 0.4 episode, p = 0.38 a non-significant difference. Hyperglycemic episodes markedly reduced after 24 hours in both arms with mean of 0.9 ± 1.0 (metformin) vs. 1.2 ± 1.0 (placebo) p = 0.15 on Day-2 and 0.6 ± 1.0 (metformin) vs. 0.7 ± 1.0 (placebo) on Day-3 p = 0.67. Few hypoglycemic episodes were seen after 24 hours in both arms with mean of 0.7 ± 0.9 (metformin) vs. 0.6 ± 0.8 (placebo) on Day-2 and 0.8 ± 0.9 (metformin) and 1.0 ± 0.8 (placebo) on day-3.

**Table 2:** Primary outcome and similar secondary glycemia based outcomes after randomization to metformin or placebo

	Metformin	Placebo	*p* value
**Primary outcome**			
**First 24 hours**	*n* = 47^a^	*n* = 49^a^	
Hyperglycemic^b^ episodes	3.9 ± 1.4	4.1 ± 1.6	0.64
	4 [3-5]	4 [3-5.5]	0.49
**Secondary outcomes**			
**First 24 hours**			
Hypoglycemic^c^ episodes	0.1 ± 0.2	0.1 ± 0.4	0.38
	0 [0–0]	0 [0–0]	0.49
**Second 24 hours**	*n* = 46^a^	*n* = 40^a^	
Hyperglycemic^b^ episodes	0.9 ± 1.0	1.2 ± 1.0	0.15
	1 [0–2]	1 [0–2]	0.13
Hypoglycemic^c^ episodes	0.7 ± 0.9	0.6 ± 0.8	0.68
	0 [0–1]	0 [0–1]	0.76
**Third 24 hours**	*n* = 37^a^	*n* = 37^a^	
Hyperglycemic^b^ episodes	0.6 ± 1.0	0.7 ± 1.0	0.67
	0 [0–1]	0 [0–1]	0.35
Hypoglycemic^c^ episodes	0.8 ± 0.9	1.0 ± 0.8	0.42
	1 [0-1.5]	1 [0–1]	0.38
**Day 1 to 3**	*n* = 37^d^	*n* = 35^d^	
Hyperglycemic^b^ episodes	5.3 ± 2.0	5.8 ± 2.2	0.32
	5 [4-7]	6 [5-7]	0.28
Hypoglycemic^c^ episodes	1.6 ± 1.4	1.7 ± 1.4	0.85
	2 [0–3]	1 [1-2]	0.94
Severe hypoglycemic^e^ episodes	0.03 ± 0.16	0.14 ± 0.43	0.14
	0 [0–0]	0 [0–0]	0.15
**Day 1 to 3**	*n* = 48^f^	*n* = 50^f^	
Any hyperglycemia^b^	48 (100)	50 (100)	0.51
Any hypoglycemia^c^	30 (63)	34 (68)	0.56
Any severe hypoglycemia^e^	1 (2)	4 (8)	0.18

Data expressed as mean ± standard deviation, median [interquartile range] and number (%). Analyses by Student t test for means, Mann-Whitney u test for medians and Chi Square test for categorical data sets

^a^Number of monitoring compliant patients included in analysis: compliance defined as at least four of the six pre or 2-hour post meal sugar capillary blood profile points done in the relevant 24-hour block of the 3-day trial period

^b^Hyperglycemia defined as self-monitored pre meal capillary blood glucose ≥ 5.3 or 2-hour post meal ≥ 6.7 mmol/L

^c^Hypoglycemia defined as any self-monitored capillary blood glucose ≤ 3.9 mmol/L

^d^Only participants classified as compliant over all 3 trial days included

^e^Severe hypoglycemia defined as capillary blood glucose < 3 mmol/L

^f^Participants with at least one capillary glucose reading within trial period included

Table 3 shows the other secondary outcomes. A total of 4 participants (all GDM cases) required unplanned anti-glycemic agent, 3/49 (6.1 %) in metformin arm and 1/50 (2.0 %) in placebo arm in the form of open labeled metformin. Mild side effects of nausea (4.0 %), vomiting (2.0 %) and diarrhea (6.0 %) were seen in metformin treated arm. All other secondary outcomes for both mother and neonate were not significantly different.

**Table 3:** Other secondary trial outcomes

Outcome	Metformin	Placebo	*p* value
Open label antiglycemic agent^a^	3/49 (6 %)	1/50 (2 %)	0.36
Nausea^b^	2/49 (4 %)	0/50 (0 %)	0.24
Vomiting^b^	1/48 (2 %)	0/50 (0 %)	0.49
Diarrhea^b^	3/48 (6 %)	1/50 (2 %)	0.36
**Mode of delivery**			
*Spontaneous vertex delivery*	19/52 (37 %)	10/51 (20 %)	0.16
*Vacuum/Forceps delivery*	4/52 (8 %)	5/51 (9.8 %)	
*Cesarean delivery*	29/52 (56 %)	36/51 (71 %)	
	*n* = 52	*n* = 51	
Estimated blood loss (ml)	300 [213–475]	300 [300–400]	0.80
	*n* = 48	*n* = 50	
VNRS SMBG^c^	9 [8-10]	9 [8-10]	0.46
VNRS Allocated Medicine^d^	9 [1-10]	9 [1-10]	0.23
	*n* = 52	*n* = 51	
Birth weight (kg)	2.51 ± 0.61	2.66 ± 0.56	0.21
	*n* = 52	*n* = 51	
Apgar Score at 1 minute	9 [9]	9 [9]	0.68
Apgar Score at 5 minutes	10 [10]	10 [10]	0.23
	*n* = 46	*n* = 50	
Umbilical arterial blood pH	7.30 [7.24–7.35]	7.32 [7.25–7.35]	0.43
	*n* = 45	*n* = 47	
Umbilical arterial base deficit	2.1 [0.7–4.4]	1.8 [0.8–3.7]	0.52
SCN/NICU^e^ Admission	18/52 (35 %)	14/51 (28 %)	0.43
**Indication for NICU admission**			
* Prematurity*	6 (33.3 %)	4 (28.6 %)	0.59
*Transient tachypnea of newborn*	5 (27.8 %)	2 (14.3 %)	
*Presumed sepsis*	2 (11.1 %)	4 (28.6 %)	
*Others*^*f*^	5 (27.8 %)	4 (28.6 %)	

Data expressed as number (%), mean ± standard deviation, median [interquartile range] and number (%). Analyses by Student t test for comparisons of means, Mann Whitney U test for comparison of non-parametric or ordinal data, Chi square test for categorical datasets (Fisher’s exact test for 2 × 2 categorical datasets if any cell < 5). All tests 2-sided

^a^Metformin or insulin if BSP > 11 mmol/L or at providers’ discretion for severe hyperglycemia

^b^Any reported episode recorded during the 3-day trial

^c^Visual numerical rating scale (scored 0–10, higher score greater satisfaction) for experience with self-monitoring of capillary blood glucose

^d^ Visual numerical rating scale (scored 0–10, higher score greater satisfaction) for experience with allocated medicine

^e^Special care nursery/Neonatal intensive care unit

^f^ Others: neonatal jaundice 5, respiratory distress syndrome 2, duodenal atresia 1 and suspected hypoxic ischemic encephalopathy 1

**Fig. 2:**
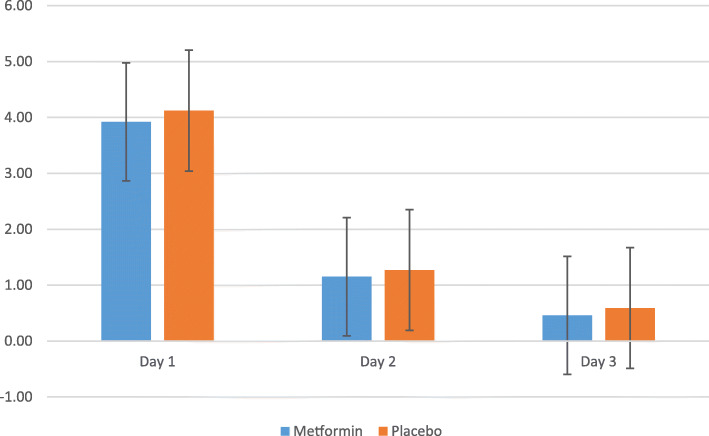
Mean hyperglycemic episode day 1–3 after randomization to metformin or placebo

**Fig. 3:**
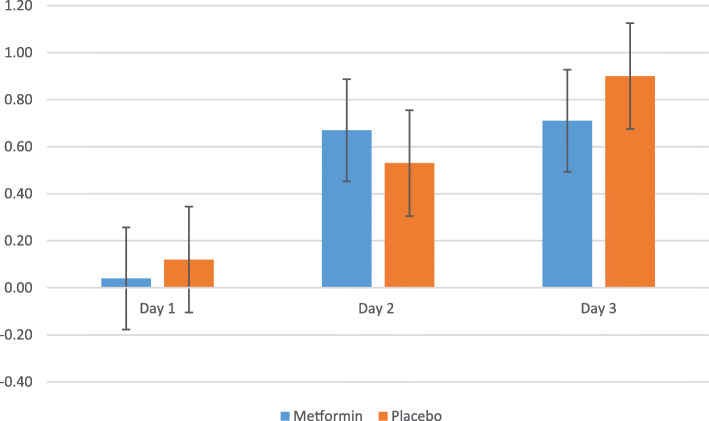
Mean hypoglycemic episode day 1–3 after randomization to metformin or placebo

The hyperglycemic episodes after antenatal dexamethasone demonstrated in this study were mild and transient as shown in Fig. 2. Mild hyperglycemia was mainly on day 1 and with return to euglycemia on day 2 and 3. On the other hand, hypoglycemic episodes were very few from day 1 to 3 with no significant difference in both arms (Fig. 3).

### Post hoc analyses

Post hoc analysis looking into mean hyperglycemic episodes from fourth to ninth (from 12-hours after study drug administration) BSP readings showed no significant difference 2.7 ± 1.5 (metformin) vs. 3.1 ± 1.4 (placebo) *p* = 0.16 (Table 4). We also looked in subgroup analyses stratified according to GDM status (Table 4) with regard to hyperglycemic episodes on Day-1; amongst women without GDM 3.9 ± 1.4 vs. 4.0 ± 1.7 *p* = 0.80 and GDM 3.9 ± 1.5 vs. 4.4 ± 1.6 *p* = 0.40 for metformin and placebo arms respectively. This analysis was not powered. As metformin did not appear to significantly impact glycemic response in this trial, we analyzed the impact of ACS according to GDM status incorporating the entire trial population on Day-1 hyperglycemic episodes which were 4.2 ± 1.6 vs. 4.0 ± 1.5 *p* = 0.53 indicating that ACS impact was very similar for women without GDM and milder GDM cases controllable on lifestyle change.

**Table 4:** Post-hoc analysis on main outcomes

	Metformin *n* = 52	Placebo *n* = 51	*p* value
**Primary outcome**			
**First 24 hours**	*n* = 47^a^	*n* = 49^a^	
Hyperglycemia episodes^b^			
Non GDM	3.9 ± 1.4	4.0 ± 1.7	0.81
GDM	3.9 ± 1.5	4.4 ± 1.6	0.40
**Secondary outcomes**			
**First 24 hours**			
Hypoglycemia episodes^c^			
Non GDM	0.1 ± 0.2	0.2 ± 0.5	0.23
GDM	0 ± 0	0.0 ± 0.0	0.48
**Second 24 hours**	*n* = 46^a^	*n* = 40^a^	
Hyperglycemia episodes^b^			
Non GDM	1.0 ± 1.3	1.1 ± 1.0	0.83
GDM	1.5 ± 1.0	1.8 ± 1.2	0.70
Hypoglycemia episodes^c^			
Non GDM	0.8 ± 0.9	0.6 ± 0.8	0.48
GDM	0.4 ± 0.9	0.3 ± 0.6	0.27
**Third 24 hours**	*n* = 37^a^	*n* = 37^a^	
Hyperglycemia episodes^b^			
Non GDM	0.5 ± 1.0	0.6 ± 0.8	0.94
GDM	0.3 ± 0.7	0.7 ± 1.1	0.61
Hypoglycemia episodes^c^			
Non GDM	0.7 ± 0.9	0.8 ± 0.8	0.37
vGDM	0.9 ± 0.8	1.1 ± 0.9	0.43
**Blood sugar profile points 4–9**			
Hyperglycemic episodes	2.7 ± 1.5	3.1 ± 1.4	0.16
	3 [2-4]	3 [2-4]	0.15

Data expressed as mean ± standard deviation and number (%). Analyses by Student t test for comparison of means. P > 0.05 for all analyses

^a^Number of monitoring compliant patients included in analysis: compliance defined as at least four of the six pre or 2-hour post meal sugar capillary blood profile points done in the relevant 24-hour block of the 3-day trial period

^b^Hyperglycemia is defined as pre meal blood glucose level of ≥ 5.3 mmol/L and 2 hours post prandial/meal blood glucose of ≥ 6.7 mmol/L

^c^Hypoglycemia is defined as capillary blood glucose level ≤ 3.9 mmol/L

No major harms (maternal ICU admission, diabetic ketoacidosis and perinatal mortality) occurred to participants or their offspring within the trial.

## Discussion

The results of this study indicate that antenatal corticosteroids administration causes universal hyperglycemia (at least one hyperglycemic episode seen in all participants). The effect was mild and transient. Our finding on duration is in contrast to a study on antenatal betamethasone that showed mean maximum blood glucose were higher for those with diabetes (11.3 vs. 9.7mmol/L, *p*⩽0.01) with mean time to reach the maximum glucose level similar for both groups at 30 hours [8]. For insulin dependent diabetic pregnancies after intramuscular betamethasone, hyperglycemia peaks on day-2 and − 3 lasting up to day-5 [9].

Our data demonstrated very few hypoglycemic episodes on day-1 to 3 (Fig. 3) in both trial arms. The hypoglycemic episode showed no significant difference and metformin does not cause more hypoglycemia as compared to placebo. Metformin reduced fasting serum insulin by 40 % hence the risk of hypoglycemia is minimal [19].

Post hoc analyses of mean hyperglycemic episodes from fourth to ninth self-blood glucose readings were done, in order to account for the pharmacokinetic impact from initiation of metformin. The onset of metformin is about three hours with peak plasma concentrations (C_max_) reached within one to three hours of taking it [20]. However, there were no significant differences between the two arms. Although the mean hyperglycemic episodes between metformin vs. placebo arm was marginally larger (difference of 0.4), the difference was not significant.

In our trial, prophylactic metformin was not able to ameliorate the hyperglycemic effect especially during the first 24 hours in contrast to a study by Star et al., which demonstrated that prophylactic insulin significantly reduced the degree of hyperglycemia, although the doses used in their trial did not entirely eliminate the hyperglycemic effect of corticosteroids [6]. Insulin may be a better treatment due to the pharmacokinetics of metformin with an average elimination half-life in plasma of 6.2 hours, 90 % of the drug is cleared within 24 hours in patients with normal renal function [24]. This is particularly true, as our study population showed hyperglycemia mainly in the first 24 hours after antenatal dexamethasone administration. Mathiesen et al. recommends an algorithm for fetal lung maturation in diabetic women to prevent severe dysregulation of metabolic control [9].

Our trial has strengths and limitations. To our best knowledge, this is an original and first study to report on giving prophylactic metformin after antenatal corticosteroids. Previous studies were on insulin prophylaxis or treatment [6, 10]. This is a randomized, double-blind and placebo-controlled trial with a clear criterion of selecting women in the lower end of risk of hyperglycemia following antenatal corticosteroids. We had few women who declined to participate, no cross over and complete outcome data. Participants were confident to perform self-blood glucose monitoring and found it acceptable, as it did not require them in-patient monitoring.

The limitation of our study, which is the sample size, although powered for surrogate outcome (maternal hyperglycemia), while the more relevant outcome would be to prevent fetal acidosis. Hence, for this outcome the study has insufficient power. The metformin dose used in our study (500 mg twice a day) is lower than the maximum dose which might not be sufficient to eliminate hyperglycemia after ACS. This lower dose was used due to its potential gastrointestinal side effects.

## Conclusions

In women with and without mild GDM, antenatal corticosteroids cause maternal hyperglycemia primarily on day one only. The effect is fairly mild. Prophylactic metformin after antenatal corticosteroids does not reduce its hyperglycemic effect.

Funding information: Internally funded by University of Malaya (Department of Obstetrics & Gynecology).

## Data Availability

All data generated or analyzed during this study are included in this published article and the datasets used are available from the corresponding author on reasonable request and subject to approval of our ethics board.

## Abbreviations

ACS: Antenatal corticosteroids
BSP: Blood sugar profile
GDM: Gestational diabetes mellitus
